# Case report: Development of anxiety symptoms after receiving the Pfizer-BioNTech (BNT162b2) COVID-19 vaccine: a case series

**DOI:** 10.3389/fpsyt.2024.1514428

**Published:** 2025-01-22

**Authors:** Maris Taube, Alise Alma Lesiņa

**Affiliations:** ^1^ Department of Psychosomatic Medicine and Psychotherapy, Riga Stradiņš University, Riga, Latvia; ^2^ Department of Depression and Crisis, National Centre of Mental Health, Riga, Latvia; ^3^ Faculty of Medicine, Riga Stradiņš University, Riga, Latvia

**Keywords:** COVID-19 vaccines, SARS-CoV-2 vaccine, BioNTech162 vaccine, anxiety, case reports

## Abstract

Severe acute respiratory sindrome - Coronavirus - 2 (SARS-CoV-2) (Coronavirus disease - 19 (COVID-19)) infection can result in long-term health consequences, such as long COVID. The clinical manifestations of long COVID include depression, anxiety, brain fog with cognitive dysfunction, memory issues, and fatigue. However, the links between vaccination and psychiatric disorders have been less studied. This article describes three patients who reported anxiety after receiving a complete course of the Pfizer-BioNTech BNT162b2 vaccine. It is important to explore the relationship between anxiety, other mental health disorders, and COVID-19 vaccination, as well as to investigate potential pathogenetic mechanisms.

## 1 Introduction

Currently, the persisting symptoms of SARS-CoV-2 (COVID-19) infection have received considerable clinical interest (1), with research focusing on both the early and later stages of the COVID-19, including long-lasting symptoms in some patients (2, 3). The inflammatory process, particularly cytokine storms, has also attracted significant attention, and researchers are currently investigating the persistence of symptoms in COVID-19 patients (4). The selective serotonin reuptake inhibitors (SSRIs), a group of antidepressant medications, have been reported to alleviate clinical symptoms observed during COVID-19 infection (5). However, in addition to examining the history and therapeutic approaches used to treat long COVID, the possibility of serotonin dysregulation (6) should be considered. An imbalance of the biogenic amine neurotransmitter serotonin is one of the primary hypotheses underlying the causes of anxiety and depression. However, the impact of vaccines on psychiatric symptoms remains unclear.

Various studies have shown that all approved COVID-19 vaccines are effective, safe, and immunogenic (7). Additionally, cohort studies examining the impact of COVID-19 vaccination on mental health have revealed increased risks of depression, anxiety, dissociative disorders, stress-related and somatoform disorders, as well as sleep disorders. Conversely, a reduction in the incidence and risk of schizophrenia and bipolar disorder has also been observed (8). However, psychiatric adverse effects associated with COVID-19 vaccination have not been sufficiently studied, and there is limited clarity regarding the impact of vaccines on psychiatric symptoms. In this report, we present a case series of three patients who reported experiencing anxiety after completing the vaccination schedule with the Pfizer-BioNTech BNT162b2 vaccine (9).

## 2 Case presentation

The inpatient unit for depression and crisis at the National Centre for Mental Health, which serves approximately 800,000 residents of Riga, the capital of Latvia, admitted three patients within a single month. These patients, all women aged 55 to 73, self-reported developing symptoms of anxiety and depression after receiving the Pfizer-BioNTech (BNT162b2) COVID-19 vaccine. The time between vaccination and the next hospitalization varied among the three patients (6, 16, and 33 months). Each patient had recovered from COVID-19 with no lasting consequences and had received both the Pfizer-BioNTech (BNT162b2) COVID-19 vaccine and a booster dose, after which anxiety and depression developed within 24 h. The number of episodes of depression and anxiety also varied between the cases.

## 3 Background history

Patient I is a 73-year-old woman of Latvian ethnicity, with primary education, from the Riga region. She reported experiencing episodic sleep disturbances and mild anxiety prior to the pandemic, which were diagnosed in 2018.

Patient II is a 67-year-old woman of Russian ethnicity, with secondary education, from the Riga region. She has undergone three hospitalizations. Prior to her postvaccination episode of anxiety and depression, she experienced two depressive episodes in 2012 and 2018, although inpatient therapy was not necessary for either of those previous episodes.

Patient III is a 55-year-old woman of Latvian ethnicity, with secondary education, from the Riga region. She reported a family history of psychiatric illness and a head injury at the age of 20. She has struggled with alcohol abuse and displayed a sluggish, deliberate manner. She began treatment for depression in 2009 after consulting a psychotherapist. In 2021, she experienced the first of four depressive episodes, all of which required hospitalization.

The anamnestic data for all cases are presented in **Table 1**.

**Table 1:** Primary anamnestic information (vaccination, main symptoms, SARS-CoV-2 infection date, psychiatric diagnosis, hospital treatment episodes, medications given, and comorbidities) for the three patients.

Patient/age/sex	Vaccination/revaccination dates	Vaccine/dosage	Main symptoms after vaccination	Time of symptom onset after vaccination	SARS-CoV-2 infection	Psychiatric diagnosis	Hospital treatment episodes	Evolution of patients after out/in-patient treatment of anxiety triggered by vaccination (CGI-I)	Main psychotropic medication used for treatment, current hospitalization	Additional health problems
Patient I/73/female	10 June 2021/1 July 2021	Pfizer-BioNTech (BNT162b2)/0.3	Anxiety, social isolation	24 h	22 February 2022 (approved)	Generalized anxiety disorder F41.1	(1) 18 January 2024–16 February 2024	3	Tianeptine, trazodone	
Patient II/67/female	4 May 2021/25 May 2021	Pfizer-BioNTech (BNT162b2)/0.3	Weakness, sleep disturbances, anxiety, depression	24 h	16 January 2022 (approved)	Recurrent depressive episode without somatic symptoms F33.10	(1) 20 September 2022–20 October 2022 (2) 11 January 2023–8 February 2023 (3) 19 January 2024–23 February 2024	3	Escitalopram, mirtazapine	Asymmetric enlargement of the subarachnoid spaces
Patient III/55/female	5June 2021/26 June 2021	Pfizer-BioNTech (BNT162b2)/0.3	Anxiety decreased appetite	24 h	February 2022 (not approved)	Recurrent depressive episode with somatic symptoms F33.11	(1) February 2021–19 March 2021 (2) 6 January 2022–18 January 2022 (3) 5 April 2023–3 May 2023 (4) 29 February 2024–27 March 2024	4	Venlafaxine, mirtazapine	Diabetes mellitus, hypothyroidism

CGI-I, Clinical Global Impression Scale-Improvement.

## 4 Treatment episodes

### 4.1 Patient I

Patient I experienced an increase in anxiety within 24 h of receiving the vaccine on 10 June 2021 and a booster on 1 July 2021. The clinical manifestations included feeling anxious, constant worrying, difficulty relaxing, irritability, avoidance behaviors, chest pain, and elevated blood pressure.

The diagnosis of generalized anxiety disorder was made based on the diagnostic criteria of the International Statistical Classification of Diseases and Related Health Problems: Tenth Revision (ICD-10) (10) and the validated 7-Question Generalized Anxiety Disorder Scale (GAD-7) (11), as recommended by local clinical guidelines.

Patient I received outpatient treatment with medications (SSRI antidepressants, antihypertensives, and benzodiazepines), which led to minor clinical stabilization and a decrease in symptom intensity for about 27 months, followed by a worsening of symptoms in the last 6 months prior to hospitalization.

The patient was treated for 29 days at an inpatient unit with both pharmaceutical and nonpharmaceutical therapies (visual arts, music, theater, dance movement therapy, psychotherapy, and occupational therapy). Trazodone and tianeptine were administered to treat the patient’s anxiety, resulting in an improvement in her condition. Age-specific and nonsignificant changes were observed in subsequent examinations (computed tomography, electroencephalography, and transcranial and intracranial vessel duplex).

### 4.2 Patient II

The patient reported a significant decline in her health, along with anxiety and depression, following the administration of the Pfizer vaccine on 4 May 2021, and a booster on 25 May 2021.

After receiving the booster, the patient experienced another bout of anxiety and depression within 24 h, accompanied by persistent worrying, difficulty relaxing, restlessness, weakness, an inability to leave home due to anxiety, and loss of appetite, resulting in a weight loss of 10 kg.

The diagnosis of depressive episode without somatic symptoms was made based on applied diagnostic criteria of the ICD-10 and the validated Patient Health Questionnaire 9-Item Scale (PHQ-9) for depression and the GAD-7 for anxiety disorders, as recommended by local clinical guidelines.

The patient used antidepressants for a short period, experiencing some improvement.

In September 2022, 16 months after receiving the Pfizer vaccine, the patient was hospitalized for the first time due to increased anxiety. After 30 days of hospitalization, the patient’s condition improved with the daily administration of 20 mg of escitalopram.

The patient was re-hospitalized in 2023 due to tremors and extreme anxiety. After 28 days of hospitalization, her anxiety subsided with the daily administration of 30 mg of mirtazapine.

In 2024, the patient was hospitalized for a third time, and electroencephalography revealed the presence of encephalopathy.

### 4.3 Patient III

The patient attributed her second episode of depression and the onset of anxiety to receiving the vaccine on 5 June 2021, followed by the booster on 26 June 2021.

After receiving the COVID-19 vaccine (vaccine 2), the patient developed anxiety. The symptoms appeared within 24 h and included persistent worry, difficulty relaxing, irritability, restlessness, shaking of hands, and chest pain.

The diagnosis of recurrent depressive episode without somatic symptoms was made based on the diagnostic criteria from the ICD-10 and the validated PHQ-9 for depression and the GAD-7 for anxiety disorders, as recommended by local clinical guidelines.

The patient was hospitalized 6 months after vaccination in 2022 due to severe anxiety and depression. She was treated in a hospital ward for 22 days, with a daily dosage of 225 mg of venlafaxine and 15 mg of mirtazapine, resulting in improvement of her condition.

The patient was treated for a third time in 2023 in the hospital for 20 days due to depression and anxiety, with improvement in her condition. She has been diagnosed with diabetes mellitus.

In 2024, the patient was admitted to a psychiatric hospital for the fourth time for 27 days due to depression, low mood, and suicidal thoughts. The administration of vortioxetine (20 mg) showed promising results.

### 4.4 Current status

Following their last hospitalizations, all of the patients showed improvement and decreased anxiety levels. Currently, all the patients continue to take their medication and visit a psychiatrist on an outpatient basis.

## 5 Discussion

In general, COVID-19 vaccination is not associated with psychiatric adverse events, according to the meta-analysis (12).

Nevertheless, there is new evidence suggesting a risk that the COVID-19 vaccine administered to patients with pre-existing mental illness may contribute to their hospitalization (13), potentially as an anxiety response to the act of vaccination (14). Furthermore, a depressive episode occurring prior to receiving the Pfizer-BioNTech COVID-19 vaccine is a negative predictor of antibody titers (15).

Different mechanisms may play a role in the development of anxiety, including inflammatory processes, changes in psychotropic metabolism, autoimmune responses, and stress related to the vaccination itself (16).

In all three presented cases, the patients received a full course of the Pfizer-BioNTech (BNT162b2) vaccine between May and July of 2021 and subsequently developed anxiety.

Common aspects of all three cases include the same vaccine, pre-existing psychiatric symptoms, relatively severe symptoms of anxiety, and a prolonged recovery process.

Comorbidities and life events (e.g., head trauma, substance use such as alcohol and benzodiazepines) will be important aspects to consider in future surveys.

Moreover, each patient independently and actively reported their anxiety without prompting from a doctor. However, reports linking vaccination to the development of anxiety should be assessed with caution (17).

### 5.1 Limitations

The underlying cause of anxiety in these cases may not be connected to the Pfizer-BioNTech (BNT162b2) vaccination course, and no strong similarities were identified between the three cases. Patients may have attributed any health problems they experienced to either COVID-19 or the vaccination, without regard to evidence.

## 6 Conclusion

There may be a connection between the Pfizer-BioNTech (BNT162b2) COVID-19 vaccine and the development of anxiety symptoms in patients with pre-existing mental health problems. The potential underlying mechanism warrants further investigation.

## Data Availability

The original contributions presented in the study are included in the article/supplementary material. Further inquiries can be directed to the corresponding author.
